# Persistent Left Superior Vena Cava: Implications of Surgical Management

**DOI:** 10.1177/2324709619855754

**Published:** 2019-06-14

**Authors:** Robin Boyer, Ramanjeet Sidhu, Aslan Ghandforoush, Theingi Win, Arash Heidari

**Affiliations:** 1 UCLA—Kern Medical, Bakersfield, CA, USA

**Keywords:** superior vena cava, coronary, transplant, surgical management

## Abstract

Persistent left superior vena cava is the most common congenital anomaly of thoracic venous return, which results when the left anterior cardinal vein fails to regress. A 41-year-old African American male with a history of an unspecified childhood cardiac murmur presented to the emergency department with congestive heart failure exacerbation revealing an incidental finding of a persistent left superior vena cava. Ultimately, he required implantable cardioverter defibrillator placement and cardiac transplantation assessment. In the setting of advanced device placement or cardiac transplantation, a persistent left superior vena cava warrants several important clinical considerations at a center capable of addressing the possibility of a right-sided approach and transplantation irregularities.

## Introduction

Persistent left superior vena cava (PLSVC) is a cardiac malformation that results when the left anterior cardinal vein fails to regress.^1^ With a reported prevalence of 0.5% in the general population, it is the most common congenital anomaly of thoracic venous return.^2^ In approximately 80% to 90% of cases, the PLSVC drains into the right atrium via the coronary sinus and is of no hemodynamic consequence. It is often found incidentally during cardiovascular imaging or surgery.^1^

Diagnostic methods to identify a PLSVC include transesophageal or transthoracic echocardiography, conventional contrast venography, computed tomography, and magnetic resonance venography.^3^ It has been reported that PLSVC is identified more frequently in patients undergoing advanced device placement.^1,4^ Presence of a PLSVC may complicate pacemaker or implantable cardioverter defibrillator (ICD) placement and cardiac transplantation. In this article, we present the case of a 41-year-old male with an incidental finding of PLSVC ultimately requiring ICD placement and cardiac transplantation assessment.

## Case Report

A 41-year-old African American male presented to the emergency department with orthopnea, new-onset scrotal swelling, and bilateral lower extremity edema. His medical history was significant for unspecified childhood cardiac murmur, hypertension, and severe congestive heart failure, with reduced ejection fraction of 15% diagnosed 4 years prior to admission.

On initial presentation, the patient was afebrile, normotensive (102/72 mm Hg), with a normal cardiac and respiratory rate. His oxygen saturation was 98% on room air. Physical examination was significant for jugular venous distention approximately 13 cm H_2_O. Laboratory investigation revealed an elevated creatinine (1.62 mg/dL) above his baseline (1.05 mg/dL) collected 2 months prior, hyperkalemia (5.0 mmol/L), and hypoalbuminemia (2.3 g/dL). Cardiac troponins were negative. Electrocardiogram indicated normal sinus rate and rhythm, while chest radiography was suggestive of increased pulmonary congestion. Presentation was consistent with heart failure exacerbation and cardiorenal syndrome.

Medical management was initiated with intravenous diuretics and follow-up imaging. The patient began 60 mg of intravenous furosemide administered twice daily with a net goal to diurese 1.5 L daily. Following treatment, the patient reported a decrease in scrotal swelling and lower extremity edema. Repeated laboratory testing demonstrated an improvement in creatinine from 1.62 to 1.30 mg/dL.

Cardiology was consulted to obtain a current transthoracic echocardiogram, which revealed a severely dilated left and right ventricle, global hypokinesis with an estimated ejection fraction of 15% to 20%. The patient was noted to have grade 2 diastolic dysfunction as well as prominent left ventricular (LV) trabeculae concerning for LV non-compaction. Additionally, an abnormality concerning for a dilated coronary sinus versus possible aneurysm of the left circumflex artery was identified. An outpouring structure at the inferior left atrium was suspicious of a PLSVC warranting further imaging.

**Figure 1.**
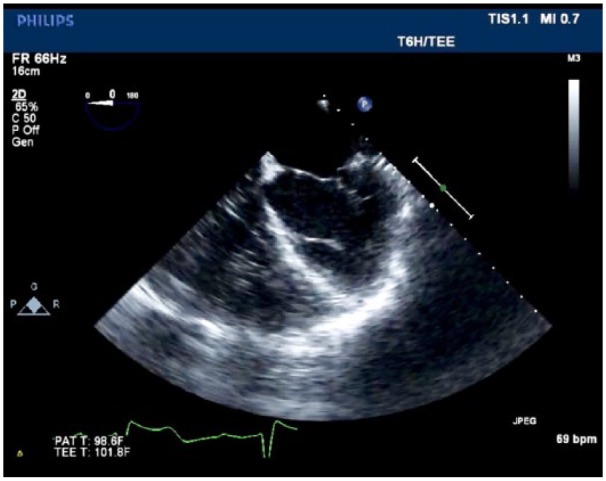
TEE (transesophageal echocardiogram) with bubble study confirming the presence of a persistent left superior vena cava.

Cardiology recommended transesophageal echocardiography with bubble study as the preferred imaging technique as contrast venography may compromise renal function. Additionally, his treatment plan was to include ICD placement for primary prevention of sudden cardiac death. The following day, the patient underwent subsequent transesophageal echocardiography with bubble study, which confirmed the presence of a PLSVC (Figure 1, Supplemental material).

At this time, right heart catheterization was performed indicating severe pulmonary hypertension, a pulmonary capillary wedge pressure of 65 mm Hg, with a decreased cardiac output and index, 2.13 and 1.1, respectively, necessitating cardiac transplant consideration. He was medically managed with a stable hospital course awaiting ICD placement and transplantation assessment.

Our patient required a facility with transplantation experts who may recognize and prepare for irregularities that may occur during procedures. If our patient was deemed to have decreased venous return during bypass or inadequate innominate vein anatomy, he would be at an increased risk for complications. Additionally, a right-sided approach for ICD placement would be preferential. Ultimately, the patient was transferred to a tertiary care center for ICD placement and cardiac transplantation assessment, as our institution does not have the expertise needed to evaluate and treat this condition.

## Discussion

Our patient highlights the important considerations of surgical management in PLSVC requiring ICD placement and cardiac transplantation assessment.

Clinical implications are encountered when the left subclavian vein is used to access the right side of the heart or pulmonary vasculature. When advanced device placement such as permanent pacemaker or ICD is warranted, a right subclavian vein approach should be performed. This is important to note in our patient as he is pending ICD placement. Access achieved with the left subclavian may result in incorrect positioning during central venous cannulation or pacemaker implantation.^5^ Inadvertent placement complications include cardiogenic shock, arrhythmias, cardiac perforation or tamponade, and coronary sinus thrombosis.^1,6^ Fortunately, a review of the literature reports a relatively low incidence of such complications.^1^

When assessing for cardiac transplantation in a patient with PLSVC, clinicians must determine the level of venous return during bypass as well as the bicaval anastomosis. The development of the innominate vein should be noted prior to cardiac transplantation. Occluding the left superior vena cava to yield acceptable venous drainage is surgically appropriate with a well-developed innominate vein. However, an inadequate or absent innominate vein increases the risk of neurovascular complications if the left superior vena cava is ligated.^6^ Our patient has a well-developed innominate vein allowing for left superior vena cava occlusion.

During cardiac transplantation, retrograde cardioplegia solution induces cardiac stasis while protecting the myocardium. In the presence of PLSVC, administering cardioplegia will result in inadequate myocardial protection following retrograde perfusion up the left superior vena cava.^7^ This relative contraindication may be eliminated if the PLSVC is clamped prior to solution administration.^1,7^

Surgical techniques to preserve a PLSVC during cardiac transplantation include direct anastomosis, anastomosis using a conduit, end-to-side anastomosis of the left superior vena cava to the right superior vena cava, or anastomosis of both right and left superior vena cava to the innominate vein of the donor graft.^6,8^ Harvesting greater lengths of donor superior vena cava and innominate vein allows for easier anastomosis of the PLSVC to the right atrial compartment.^9^ Similar to our patient, Rabago and colleagues successfully performed an orthotopic heart transplant in a patient with PLSVC and isolated LV non-compaction using a bicaval anastomosis technique. The native coronary sinus was delicately isolated to allow the left superior vena cava drain into the native inferior vena cava.^10^ This careful dissection of the coronary sinus is necessary to permit re-anastomosis.^6^ Due to comparability, a bicaval technique would likely yield similar positive results and should be considered in our patient requiring cardiac transplantation.

## Conclusion

Although considered benign in isolation, PLSVC is of clinical significance when attempting right-sided cardiac access, cardiopulmonary bypass, and consideration of cardiac transplantation as encountered in our patient. A left subclavian approach should be avoided when attempting right-sided access. Instead, a right-sided subclavian route should be taken to avoid the challenges faced during advanced device placement as in our patient. Additionally, it is important to note the development of the innominate vein, considerations of administering retrograde cardioplegia, and method of anastomosis for an optimal surgical outcome.

## Supplemental Material

Bubble_into_LSVC – Supplemental material for Persistent Left Superior Vena Cava: Implications of Surgical Management Click here for additional data file. Supplemental material, Bubble_into_LSVC for Persistent Left Superior Vena Cava: Implications of Surgical Management by Robin Boyer, Ramanjeet Sidhu, Aslan Ghandforoush, Theingi Win and Arash Heidari in Journal of Investigative Medicine High Impact Case Reports
